# The Role of Immunological and Clinical Biomarkers to Predict Clinical COVID-19 Severity and Response to Therapy—A Prospective Longitudinal Study

**DOI:** 10.3389/fimmu.2021.646095

**Published:** 2021-03-17

**Authors:** Ana Copaescu, Fiona James, Effie Mouhtouris, Sara Vogrin, Olivia C. Smibert, Claire L. Gordon, George Drewett, Natasha E. Holmes, Jason A. Trubiano

**Affiliations:** ^1^ Centre for Antibiotic Allergy and Research, Department of Infectious Diseases, Austin Health, Heidelberg, VIC, Australia; ^2^ Clinical Immunology and Allergy, Department of Medicine, McGill University Health Center, Montréal, QC, Canada; ^3^ The Research Institute of the McGill University Health Centre, McGill University, Montreal, QC, Canada; ^4^ Department of Medicine, St Vincent’s Hospital, University of Melbourne, Fitzroy, VIC, Australia; ^5^ Department of Medicine (Austin Health), The University of Melbourne, Heidelberg, VIC, Australia; ^6^ Department of Microbiology and Immunology, The University of Melbourne, Parkville, VIC, Australia; ^7^ Department of Critical Care, Melbourne Medical School, The University of Melbourne, Parkville, VIC, Australia; ^8^ Department of Oncology, Sir Peter MacCallum Cancer Centre, The University of Melbourne, Parkville, VIC, Australia; ^9^ The National Centre for Infections in Cancer, Peter MacCallum Cancer Centre, Parkville, VIC, Australia

**Keywords:** SARS-CoV-2, interleukin-6, C-reactive protein, cytokine storm, Staphylococcus aureus bacteraemia, sepsis, acute respiratory distress syndrome

## Abstract

**Background:**

The association of pro-inflammatory markers such as interleukin-6 (IL-6) and other biomarkers with severe coronavirus disease 2019 (COVID-19) is of increasing interest, however their kinetics, response to current COVID-related treatments, association with disease severity and comparison with other disease states associated with potential cytokine storm (CS) such as Staphylococcus aureus bacteraemia (SAB) are ill-defined.

**Methods:**

A cohort of 55 hospitalized SARS-CoV-2 positive patients was prospectively recruited – blood sampling was performed at baseline, post-treatment and hospital discharge. Serum IL-6, C-reactive protein (CRP) and other laboratory investigations were compared between treatment groups and across timepoints. Acute serum IL-6 and CRP levels were then compared to those with suspected COVID-19 (SCOVID) and age and sex matched patients with SAB and patients hospitalized for any non-infectious condition (NIC).

**Results:**

IL-6 was elevated at admission in the SARS-CoV-2 cohort but at lower levels compared to matched SAB patients. Median (IQR) IL-6 at admission was 73.89 pg/mL (30.9, 126.39) in SARS-CoV-2 compared to 92.76 pg/mL (21.75, 246.55) in SAB (p=0.017); 12.50 pg/mL (3.06, 35.77) in patients with NIC; and 95.51 pg/mL (52.17, 756.67) in SCOVID. Median IL-6 and CRP levels decreased between admission and discharge timepoints. This reduction was amplified in patients treated with remdesivir and/or dexamethasone. CRP and bedside vital signs were the strongest predictors of COVID-19 severity.

**Conclusions:**

Knowledge of the kinetics of IL-6 did not offer enhanced predictive value for disease severity in COVID-19 over common investigations such as CRP and vital signs.

## Introduction

There has been increasing interest surrounding the function of interleukin-6 (IL-6) and other laboratory markers in severe acute respiratory syndrome coronavirus 2 (SARS-CoV-2) infection, including the role in predicting disease severity, monitoring response to therapy, and similarities with other cytokine storm (CS) disease states (1–4). Heterogeneity in SARS-CoV-2 study design and definitions of disease severity have limited advances in understanding the clinical implications for IL-6 and other inflammatory and clinical makers in coronavirus disease 2019 (COVID-19) (4). Further, IL-6 kinetics in SARS-CoV-2 and comparisons with other infective syndromes with CS, such as bacterial sepsis, have not been extensively undertaken.

## Methods

### Participants and Setting

Adults aged ≥ 18 years hospitalized with suspected or confirmed SARS-CoV-2, no known hypersensitivity to tocilizumab and no active pulmonary tuberculosis, were enrolled sequentially in this single-center prospective cohort study between May 15^th^ 2020 and August 21^st^ 2020 at Austin Health, Melbourne, Australia.

### Definitions

Severe disease was defined as requirement for supplemental oxygen for ≥ 24 hours and a Sp02 ≤ 94% on room air and/or admission to intensive care unit (ICU), adapted from the National Institutes of Health (NIH) criteria for disease severity (5). Treatment for COVID-19 was as per hospital approved treatment protocol. Patients that required oxygen received dexamethasone, remdesivir was utilized in those that had oxygenation < 94% and were early in disease and tocilizumab was only considered on a case by case basis in intubated patients with evidence of cytokine storm (4) and no occult sepsis (negative procalcitonin). A 5-day course of remdesivir and/or dexamethasone for up to 10 days was administered to patients with severe disease starting on the 23^rd^ of June 2020.

### Objectives

The primary objective of the study was to describe the kinetics of IL-6 and other biomarkers during SARS-CoV-2 infection. Secondary objectives were to describe the association of these markers with (1) disease severity (defined as ICU admission, oxygen therapy or a composite of both), (2) treatment with dexamethasone and/or remdesivir, and (3) other disease states associated with CS such as *Staphylococcus aureus* bacteraemia (SAB).

### Data Collection and Cohorts

**Figure 1:**
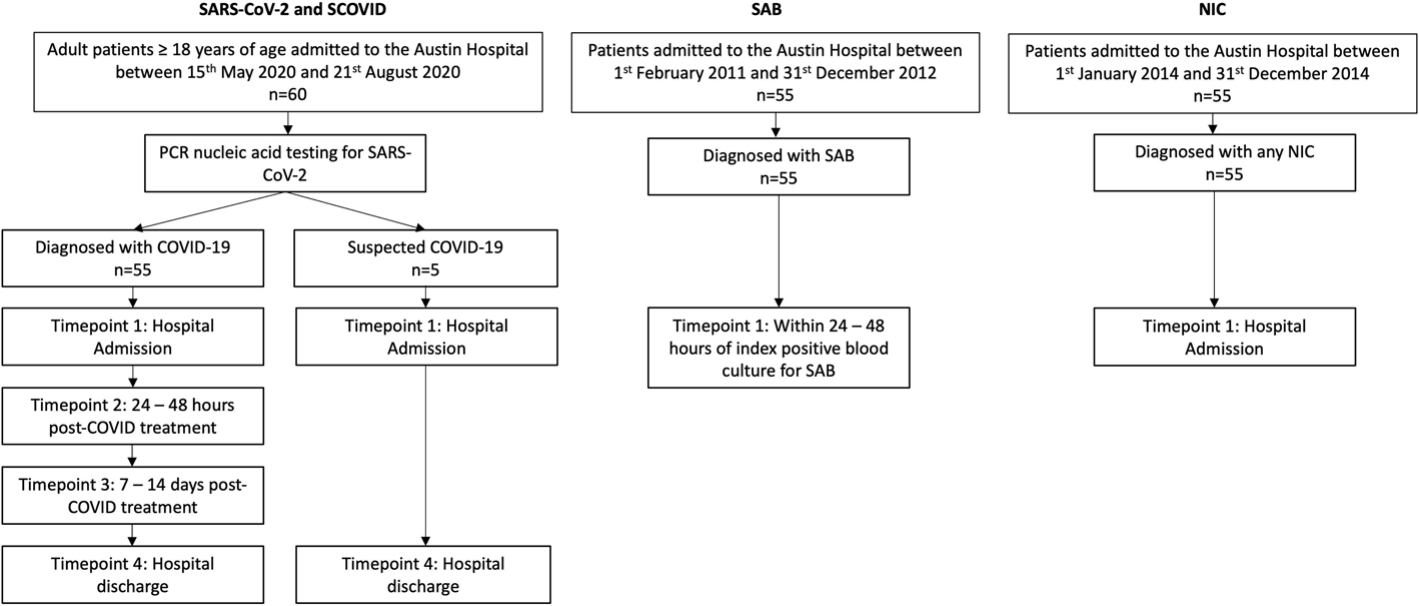
Study design outlining patient cohorts and time points for sample collection. SAB and NIC cohorts are age and sex matched with the SARS-CoV-2 cohort. PCR, polymerase chain reaction; NIC, non-infectious condition; SAB, Staphylococcus aureus bacteraemia; SARS-CoV-2, severe acute respiratory syndrome coronavirus.

Standard baseline demographic and clinical characteristics, laboratory parameters (including C-reactive protein (CRP), lymphocyte count, ferritin, lactate dehydrogenase (LDH) and D-dimer) and COVID-related treatment data (requirement for oxygen therapy and/or intubation, drug therapy with dexamethasone and/or remdesivir) were gathered. Serum samples were collected at four timepoints: (1) hospital admission, (2) 24 to 48 hours post dexamethasone and/or remdesivir, (3) 7 to 14 days post-treatment, and (4) discharge (**Figure 1**).

We compared the SARS-CoV-2 group with three distinct inpatient cohorts with potentially varied cytokine storm disease states: (1) patients with SAB; (2) patients hospitalized for any non-infectious conditions (NIC); and (3) patients with suspected COVID-19 (SCOVID) concurrently recruited with the SARS-CoV-2 patients from Austin Hospital. The patients with SCOVID had a minimum of 2 negative COVID-19 tests upon hospital admission as well as repeated testing depending on their clinical evolution. The SARS-CoV-2 patients were age and sex matched with previously described SAB (6) and NIC (7) cohorts (**Figure 1**). No other demographic information was available for the patients recruited in theses cohorts. The serum samples for the SAB and NIC cohorts were stored since their initial recruitment in accordance with well-known laboratory storing procedures at -80°C ensuring adequate back-up capacity for the freezers under the supervision of trained personnel and with ongoing alarm systems designed to monitor the temperature. Serum IL-6 from each patient cohort was quantified using an enzyme-linked immunosorbent assay (ELISA) assay (Crux Biolabs®, Australia) following manufacturers’ instructions (8) and read at a wavelength of 450 nm with a FLUOstar Optima plate reader (BMG labtech®).

### Statistical Analysis

Categorical variables are reported as counts and percentages; continuous variables as medians (interquartile range, IQR). IL-6 levels across time in the treatment groups were compared using mixed effects linear regression, while linear regression was used to compare between treatment groups. The outcome was log-transformed and the results reported as exponentiated regression coefficients (95% CI). Characteristics of matched cohorts (COVID, SAB and NIC) were compared using sign-rank test and McNemar test. IL-6 levels between cohorts (SAB vs COVID and NIC vs COVID) were compared using paired t-test. Univariable logistic regression was used for evaluation of demographic, clinical and laboratory variables with supplemental oxygen requirement. Results were expressed as odds ratios (95% CI). Competing risk time to event analysis was performed to measure the association of IL-6 and CRP with oxygen supplementation. The measured treatment outcomes were admission to ICU, oxygen therapy or a composite of both. Discharge was taken as competing risk and IL-6/CRP were separately entered as admission values and as time-varying covariates. Results were reported as sub-hazard ratios (SHRs) with 95% CI. Stata/IC 16.1 was used for all analysis. The study was approved by the local human research ethics committee (Ref HREC/63201/Austin-20). All patients, their legal representatives or their next of kin provided informed consent for this study.

## Results

### Cohort Characteristics

The baseline demographics and clinical characteristics for SARS-CoV-2 positive cohort (n=55) are described in **Table 1**. The laboratory values and treatment information are listed in **Table 2**. Twenty-five (45%) of the patients required continuous supplemental oxygen for ≥ 24 hours after SpO_2_ fell below 94% on room air and 15 (27%) were admitted to ICU. Two patients died while in hospital. There were 28 patients who received dexamethasone (50.9%); 15 who received remdesivir (27.3%) and 15 (27.3%) who received both.

**Table 1:** Patient characteristics of SARS-CoV-2 positive cohort.

Characteristics	COVID (n = 55)		
	All patients (n = 55)	ICU Admission (n = 15)	Supplemental O_2_ (n = 25) ψ
Age (years), (median, IQR)	58 (40; 70)	59 (50; 69)	66 (52; 71)
Sex (M:F)	31:24	10:5	17:8
Ethnicity (no.; %)	ATSI (1; 1.8%) African (4; 7.3%) Caucasian (31; 56.4%) East Asian (4; 7.3%) Indo-Asian (2; 3.6%) Other (1; 1.8%) Unknown (12; 21.8%)	African (3; 20%) Caucasian (8; 53.3%) Indo-Asian (1; 6.7%) Other (1; 6.7%) Unknown (2; 13.3%)	African (2; 8%) Caucasian (17; 68%) East Asian (1; 4%) Indo-Asian (2; 4%) Other (1; 4%) Unknown (3; 12%)
Smoking status (no./total no.; %)	Smoker (4/50; 8%) Ex-smoker (6/50;12%) Non-smoker (40/50; 80%)	Smoker (1/13; 7.7%) Ex-smoker (1/13; 7.7%) Non-smoker (11/13; 84.6%)	Smoker (1/21; 4.8%) Ex-smoker (4/21; 19%) Non-smoker (16/21; 76.2%)
**Comorbidities** (no.; %)			
Hypertension	20; 36.4%	6; 40%	14; 56%
Cardiac disease	7; 12.7%	2; 13.3%	5; 20%
Chronic respiratory disease	13; 23.6%	5; 33.3%	9; 36%
Chronic renal or liver disease	1; 1.8%	0; 0%	0; 0%
Diabetes	15; 27.3%	5; 33.3%	10; 40%
Immunosuppression ♣	5; 9.1%	2; 13.3%	3; 12%
Pregnancy	1; 1.8%	0; 0%	0; 0%
ACEI/ARB use	10; 18.2%	2; 13.3%	7; 28%
**Clinical characteristics**			
Charlson comorbidity index ♦ (median, IQR)	1 (0;3)	1 (1;2)	2 (1;3)
COVID-MATCH65 Score Φ (median, IQR)	3.5 (2.5;5)	4 (3.5;5)	4.5 (3.5;5)
Latency presentation recruitment ▽ (days), (median, IQR)	7 (5;10)	7 (5;10)	7 (5;9)
Length of hospital stay (days) (median, IQR)	6 (3;13)	17 (9;27)	13 (6;21)
Death (no.; %)	2; 3.6%	0; 0%	2; 8%

ACEI, angiotensin-converting-enzyme inhibitors; ARB, angiotensin II receptor blockers; ATSI, Aboriginal or Torres Strait Islander; ICU, intensive care unit; SARS-CoV-2, severe acute respiratory syndrome coronavirus 2.

♣ The immunosuppression category includes patients that are known for any of the following conditions: transplant recipient, hematological or oncological malignancy (in the last 5 years), corticosteroid use of more than 10 mg prednisolone equivalent per day, connective tissue or autoimmune condition and acquired immunodeficiency syndrome.

Ψ Patients that required supplemental O_2_ continuously for more than 24 hours during their admission.

♦ The Charlson comorbidity index is age-adjusted.

Φ COVID-MATCH65 Score is a clinical decision rule internally derived that has a high sensitivity (92.6%) and NPV (99.5%) for SARS-CoV-2 and can be used to aid COVID-19 risk assessment and resource allocation for SARS-CoV-2 diagnostics. The resulting score ranges from 1 to 6.5 points with score ≤ 1 representing low risk for a positive test (9).

▽ Time from symptoms presentation and study recruitment (days).

**Table 2:** Clinical, laboratory characteristics and treatment information for SARS-CoV-2 positive patients.

	All patients (n = 55)	ICU Admission (n = 15)	Supplemental O_2_ (n = 25) Ψ
**Patient reported clinical symptoms at baseline** (no.; %)			
Fever >38°C	26; 47.3%	9; 60.0%	14; 56.0%
Malaise/myalgia	32; 58.2%	8; 53.3%	14; 56.0%
Dyspnea	42; 76.4%	14; 93.3%	22; 88.0%
Cough	36; 65.5%	9; 60.0%	15; 60.0%
Coryza	9; 16.4%	2; 13.3%	1; 4.0%
Sore throat	16; 29.1%	2; 13.3%	2; 8.0%
Diarrhea	16; 29.1%	4; 26.7%	7; 28.0%
Other	Headache (3; 0.05%)	Headache (0; 0%)	Headache (0; 0%)
	Nausea and Vomiting	Nausea and Vomiting	Nausea and Vomiting
	(2; 0.04%)	(0; 0%)	(1; 0.04%)
	Pleuritic chest pain	Pleuritic chest pain	Pleuritic chest pain
	(4; 0.07%)	(0; 0%)	(1; 0.04%)
**Vital signs – baseline at hospital admission** (median, IQR)			
Temperature (°C)	37.8 (36.7; 38.5)	38.1 (37.1; 38.7)	38.3 (37.3; 38.8)
Respiratory rate	22 (20; 30)	35 (22; 38)	28 (22; 35)
Oxygen saturation (%)	95 (92; 98)	94 (90; 96)	92 (90; 94)
Pulse rate	96 (88; 106)	100 (92; 119)	100 (88; 115)
Blood pressure (mmHg)	118/72 (107/66; 130/81)	118/78 (100/60; 130/85)	120/69 (103/64; 130/80)
**Laboratory Data – baseline** (median, IQR)			
WCC (x10^9^/L)	6 (4.4;8)	7.2 (4.4;8)	7.1 (4.3;8)
Lymphocytes (x10^9^/L)	0.8 (0.7; 1.1)	0.7 (0.5; 0.8)	0.8 (0.6; 1)
Neutrophils (x10^9^/L)	4.3 (2.9; 6)	5.8 (2.9; 6.6)	5.1 (2.9; 6.2)
Eosinophils (x10^9^/L)	0 (0;0)	0 (0;0)	0 (0;0)
Hemoglobin (g/L)	135 (123;146)	139 (125:145)	139 (127;145)
Platelet count (x10^9^/L)	202 (171;257)	194 (161;253)	194 (161;253)
Creatinine (μmol/L)	74 (60; 94)	82 (64; 95)	82 (69; 104)
Estimated GFR ♦	90 (71;90)	86 (71;90)	79 (64;90)
Sodium (mmol/L)	139 (136;141)	138 (135;141)	139 (136;141)
Potassium (mmol/L)	4.1 (3.9;4.4); N=53	4 (4;4.4); N=13	4.1 (4;4.5); N=23
Bicarbonate (mmol/L)	25 (22; 27)	25 (24;27)	25 (24;27)
IL-6 (pg/ml)	73.9 (30.9;126.39)	56.6 (21.3;108.3)	73.9 (31.1;123.2)
CRP (mg/L)	65 (19.4; 135); N=53	135 (49.1; 223); N=15	113 (44.9; 196.5); N=24
Ferritin (μg/L)	438 (167; 864); N=50	1,084 (490; 1,570); N=15	645.5 (209; 1,311); N=24
D-dimer (mg/L)	635 (473; 972); N=53	1,394 (738; 2,505); N=15	969 (680; 1,542); N=25
LDH (U/L)	278 (236; 366); N=33	402.5 (326; 525); N=10	363 (267; 525); N=15
Bilirubin (μmol/L)	8 (6.5;13); N=48	10 (7;15); N=13	8 (6;15); N=22
ALT (U/L)	28 (21;50); N=50	37 (21; 49); N=14	28.5 (20;47); N=24
AST (U/L)	65 (55;85); N=13	66 (55;189); N=3	62.5 (55;66); N=6
GGT (U/L)	42 (25;82)	63 (40;109)	55 (38;84)
Albumin (g/L)	35 (31; 37); N=53	31 (26; 36); N=15	32.5 (30; 36); N=24
**Treatment** (no.; %)			
Mechanical ventilation	5 (9.1%)	5 (33.3%)	5 (20%)
Dexamethasone	28 (50.9%)	12 (80%)	22 (88%)
Remdesivir and dexamethasone	15 (27.3%)	8 (53.5%)	13 (52%)
Intravenous antibiotics	35 (63.6%)	15 (100%)	22 (88%)
Antifungals	3 (5.5%)	3 (20%)	3 (12%)

ALT, alanine aminotransferase; AST, aspartate transaminase; CRP, C-Reactive protein; GFR, glomerular filtration rate; GGT, gamma-glutamyl transferase; ICU, intensive care unit; IL-6, Interleukin-6; LDH, lactate dehydrogenase; SARS-CoV-2, severe acute respiratory syndrome coronavirus 2; SpO2, oxygen saturation; WCC, white cell count.

Ψ Patients that required supplemental O_2_ continuously for more than 24 hours during their admission.

♦ Estimated GFR was calculated using Chronic Kidney Disease Epidemiology Collaboration (CKD-EPI), units: ml/min/1.73.

### IL-6 and Biomarker Kinetics in SARS-CoV-2-Infected Patients

**Figure 2:**
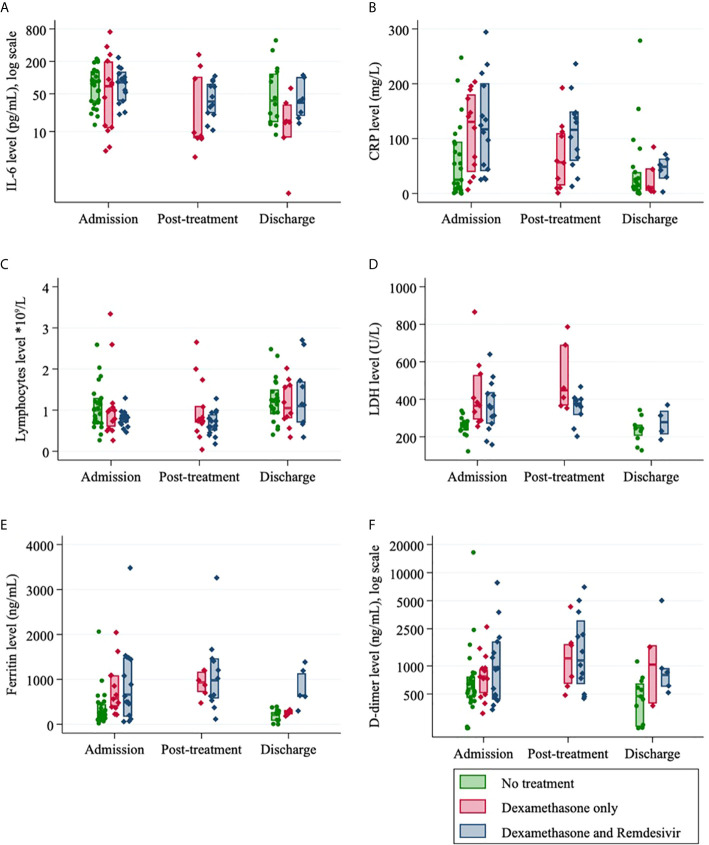
Laboratory data for SARS-CoV-2 positive patients (n=55) according to their treatment regimen and at different admission timepoints (median, IQR): **(A)** Interleukin-6; **(B)** C-Reactive protein; **(C)** Lymphocytes; **(D)** Lactate dehydrogenase (LDH); **(E)** Ferritin level and **(F)** D-dimer.

The kinetics of exploratory biomarkers over time, stratified by treatment group (dexamethasone, dexamethasone and remdesivir, no treatment) can be visualized in **Figure 2**. IL-6 and CRP values decreased with time from admission across all groups. Ferritin, LDH and D-dimer were marginally decreased from admission to discharge but increased post-treatment for the small numbers of patients in this group and lymphocytes were slightly increased from admission to discharge (**Figure 2**). At admission, there was no difference in serum IL-6 levels between those who received treatment and those who did not (**Table 3**). Post-treatment levels of serum IL-6 halved in the remdesivir and dexamethasone treatment group (p=0.023) (**Table 3**). At discharge, IL-6 was 48% lower than at admission for those who received remdesivir and dexamethasone (p=0.059) and 83% lower in the dexamethasone only group (p=0.003) (**Table 3**).

**Table 3:** Impact of COVID-19 treatment on IL-6 values.

		OR (95% CI)	p - value
**Effect of time**			
No treatment			
	Discharge vs admission	0.76 (0.47, 1.22)	0.258
Remdesivir and dexamethasone			
	Post treatment vs admission	0.52 (0.29, 0.91)	0.023
	Discharge vs admission	0.52 (0.27, 1.03)	0.059
Dexamethasone			
	Post treatment vs admission	0.54 (0.18, 1.59)	0.266
	Discharge vs admission	0.17 (0.05, 0.55)	0.003
**Comparison between groups**			
Admission			
	Treatment vs no treatment	0.91 (0.52, 1.62)	0.756
	Remdesivir (+dexamethasone vs dexamethasone	1.45 (0.54, 3.86)	0.442
Post-treatment			
	Remdesivir (+dexamethasone) vs dexamethasone	1.91 (0.62, 5.84)	0.242
Discharge			
	Treatment vs no treatment	0.45 (0.17, 1.20)	0.106
	Remdesivir (+dexamethasone) vs dexamethasone	3.03 (0.79, 11.71)	0.099

### IL-6 in SARS-CoV-2 Infected Patients Compared With Other Disease States

**Figure 3:**
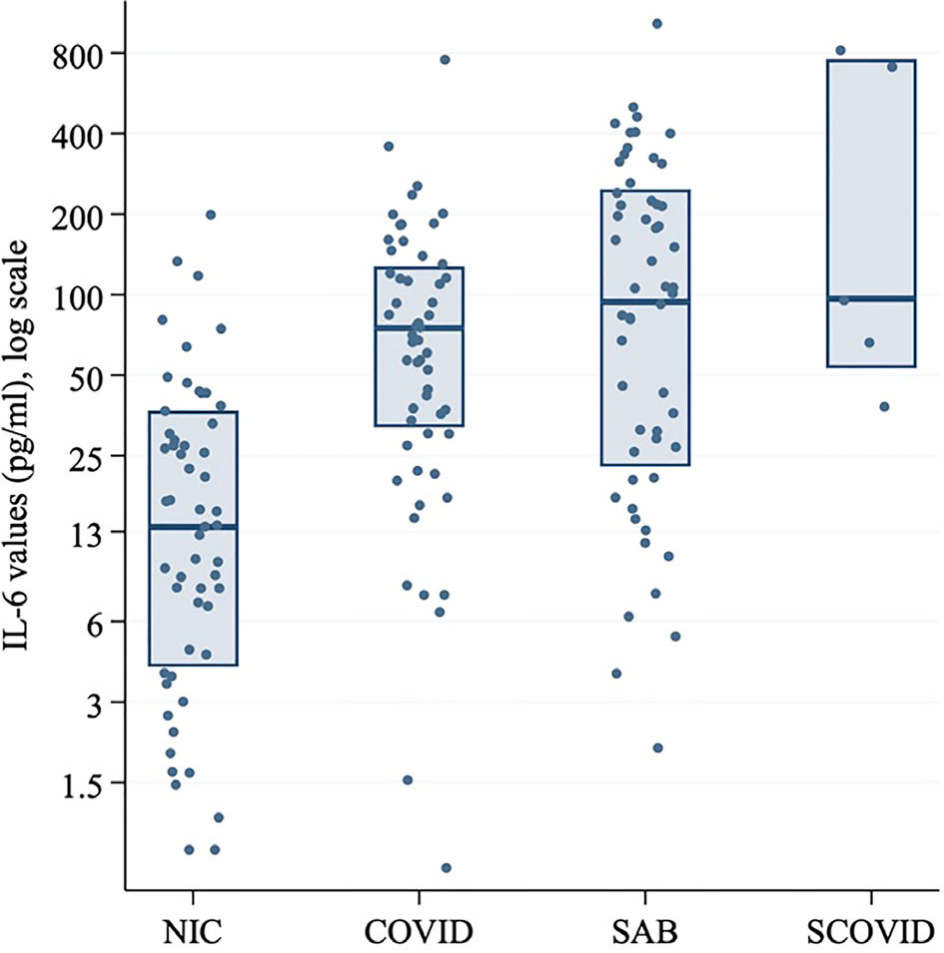
Log-transformed IL-6 values (pg/ml) for SARS-CoV-2 positive patients at baseline, SCOVID, SAB and a cohort of hospitalized patients for any NIC. The SARS-CoV-2 positive patients were age and gender matched with patients from SAB and NIC cohorts. CRP, C-Reactive protein; COVID, coronavirus disease; IL-6, Interleukin-6; LDH, Lactic acid dehydrogenase; NIC, non-infectious condition; SAB, Staphylococcus aureus bacteraemia; SARS-CoV-2, severe acute respiratory syndrome coronavirus 2; SCOVID, suspected COVID-19.

Serum IL-6 at hospital admission in SARS-CoV-2 positive patients was compared with the SCOVID, SAB and NIC groups (**Figure 3**). IL-6 values are demonstrated in **Supplementary Tables 1** and **2**. A mean difference of 72.7 pg/ml (95% CI; 40.0, 105.3) was found between the NIC and SARS-CoV-2 groups (p<0.001). Mean IL-6 was elevated in the SAB group by 57.8 pg/ml (95% CI; 0.3, 115.2) over the SARS-CoV-2 group (p=0.049). The univariable associations with ICU admission for SAB and SARS-CoV-2 positive cohorts are illustrated in **Table 4**. Although CRP was associated with ICU admission in both cohorts, IL-6 was associated with ICU admission only in the SAB cohort.

**Table 4A:** Admission characteristics and univariable model for association of ICU admission in SARS-CoV-2, Staphylococcus aureus bacteremia (SAB), SARS-CoV-2 positive and suspected COVID-19 (SCOVID) cohorts.

Factors	SAB	SARS-CoV-2 positive	p-value Ψ	NIC ♦	SCOVID
N	55	55		55	5
Female, no. (%)	25 (45%)	24 (44%)	0.564	25 (45%)	2 (40%)
Age, median (IQR)	58 (41, 70)	58 (40, 70)	0.309	58 (41, 70)	68 (54, 75)
CCI, median (IQR)	2 (0, 3)	1 (0, 3)	0.344	n/a	n/a
ICU admission	14 (25%)	15 (27%)	0.835	n/a	0 (0%)
WCC, median (IQR)	10 (6.8, 15.6)	6 (4.4, 8)	<0.001	n/a	7.6 (1.6, 10.9)
Neutrophils, median (IQR)	8.7 (5.5, 13.5)	4.3 (2.9, 6)	<0.001	n/a	155.9 (41.1, 261.5)
CRP, median (IQR)	190.9 (99.7, 290)	65 (19.4, 135)	<0.001	n/a	95.51 (52.17, 756.67)
IL-6, median (IQR)	92.76 (21.75, 246.55)	73.89 (30.9, 126.39)	0.017	12.50 (3.06, 35.77)	95.51 (52.17, 756.67)

**Ψ **The sign rank test was used for continuous values and McNemar’s test for categorical values.

**♦ **Other demographic details were not collected for this cohort. The SARS-CoV-2 positive patients were age and gender matched with patients from the SAB and NIC cohorts.

**Table 4B:** Association with ICU admission (logistic regression separately for SARS-CoV-2 positive patients and SAB).

	SARS-CoV-2 positive		SAB	
	OR (95% CI)	p-value	OR (95% CI)	p-value
Female vs male	0.55 (0.16, 1.91)	0.349	2.81 (0.80, 9.92)	0.108
Age (increase of 1 year)	1.02 (0.98, 1.05)	0.364	1.01 (0.97, 1.04)	0.625
CCI, median (IQR)	0.90 (0.59, 1.39)	0.641	0.93 (0.68, 1.27)	0.652
WCC, median (IQR)	0.99 (0.92, 1.07)	0.879	0.96 (0.86 1.7)	0.437
Neutrophils, median (IQR)	1.15 (0.94, 1.41)	0.163	0.96 (0.86, 1.08)	0.505
CRP (increase for 10 units), median (IQR)	1.13 (1.03, 1.23)	0.008	1.06 (1.00, 1.12)	0.034
IL-6 (increase for 10 units), median (IQR)	0.95 (0.87, 1.03)	0.232	1.07 (1.02, 1.12)	0.01

CCI, Charlson comorbidity index; CRP, C-reactive protein; ICU, intensive care unit; IL-6, interleukin-6; NIC, non-infectious conditions; SAB, Staphylococcus aureus bacteraemia; SARS-CoV-2, severe acute respiratory syndrome coronavirus 2; SCOVID, suspected COVID-19; n/a, non-available data; WCC, white cell count.

### Association Between IL-6 and Other Biomarkers With Clinical Outcomes in SARS-CoV-2 Infected Patients

No association between elevated baseline IL-6 and either requirement for oxygen therapy, ICU admission or composite outcomes was found on univariable analysis (**Supplementary Tables 3** and **4**). An increased CRP of 10 mg/L increased odds of oxygen therapy requirement by 13% (p=0.006), ICU admission by 1% (p=0.014) and the composite outcome by 1% (p=0.003). Using time to event analysis, IL-6 did not appear to be associated with requirement for oxygen therapy, ICU admission or a composite endpoint of both outcomes (**Table 5**). In contrast, increased CRP was still associated with oxygen therapy and the composite endpoint, even when adjusted for predictors of the outcome identified on logistic regression. After adjustment for respiratory rate and SpO_2_, an increase of 10 mg/mL in CRP was associated with a 5% increased risk of requirement for oxygen therapy (p=0.013) and a 5% increased risk of the composite outcome (p=0.025).

**Table 5:** Association of increased CRP and IL-6 values with outcome of severe disease (ICU admission, oxygen therapy or composite of both).

		N total	N with event	Unadjusted		Adjusted*	
				SHR (95% CI)	p-value	SHR (95% CI)	p-value
**ICU admission**							
	IL-6 at baseline	47	9	0.91 (0.81, 1.03)	0.144	1.01 (0.96, 1.08)	0.649
	IL-6^	47	9	1.02 (0.97, 1.07)	0.360	0.86 (0.73, 1.01)	0.064
	CRP at baseline	54	14	1.10 (1.05, 1.14)	**<0.001**	**1.05 (0.96, 1.15)**	**0.249**
	CRP^	54	14	1.09 (1.05, 1.14)	**<0.001**	**1.06 (0.98, 1.14)**	**0.149**
**Oxygen therapy**							
	IL-6 at baseline	51	21	1.00 (0.97, 1.03)	0.960	1.00 (0.96, 1.04)	0.973
	IL-6^	51	21	1.02 (0.99, 1.05)	0.270	1.03 (0.99, 1.06)	0.117
	CRP at baseline	54	24	1.08 (1.04, 1.12)	**<0.001**	**1.05 (1.00, 1.10)**	**0.047**
	CRP^	54	24	1.07 (1.03, 1.11)	**<0.001**	**1.05 (1.01, 1.10)**	**0.013**
**Composite outcome**							
	IL-6 at baseline	46	18	1.00 (0.97, 1.04)	0.807	0.99 (0.94, 1.05)	0.795
	IL-6^	46	18	1.02 (0.99, 1.06)	0.136	1.02 (0.97, 1.07)	0.448
	CRP at baseline	54	26	1.09 (1.05, 1.14)	**<0.001**	1.06 (1.01, 1.11)	**0.020**
	CRP^	54	26	1.09 (1.05, 1.13)	**<0.001**	1.05 (1.01, 1.10)	**0.025**

CRP, C-reactive protein; ICU, intensive care unit; Interleukin-6 (IL-6); SHR, sub-hazard ratio.

*Adjusted for respiratory rate (RR) (ICU admission), RR and SpO2 (oxygen therapy) and RR, SpO2 and age (composite outcome).

^Entered as a time-varying covariate. Significant unadjusted values are shown in bold font.

## Discussion

This study presents unique prospectively data with multiple time-point sampling, assessing IL-6 and other inflammatory and clinical biomarkers in response to an antiviral (i.e. remdesivir) and an immunosuppressant (i.e. dexamethasone) treatment – informing strategies to predicting clinical severity and response to therapy. IL-6 is secreted by a plethora of immune and stromal cells and exerts effects on a similarly broad array of cellular targets translating into functional pleiotropy including the synthesis of acute phase proteins in the liver, such as C-reactive protein (CRP), a surrogate for IL-6 (10, 11). CRP is frequently used in the clinical setting as a screening marker of infection and/or inflammation (12).

Although there are reports in the literature that an increase in IL-6 can correlate with disease severity in COVID-19 (4, 13), our study of a prospective SARS-CoV-2 cohort did not find that IL-6 levels offer a clinical utility for prediction of disease severity. We noted a stronger association between simple laboratory parameters (i.e. CRP) and bedside observations (i.e. SpO_2_ and respiratory rate) with disease severity, over IL-6. In our cohort, IL-6, CRP, ferritin and LDH were raised at hospital admission while lymphocytes were reduced in line with previous reports (14–16). Further, in another clinical prospective study on longitudinal immune profiling with a median of two time points of peripheral blood collection, the authors indicated an association between serum IL-6 at the time of hospital admission and the severity of COVID-19, defined based on the degree of respiratory failure (16). Similar, in a larger retrospective longitudinal study (N=317), the authors showed the same pattern of increased inflammatory markers within the initial 24 hours after admission as described in our study and previously described study and correlation with disease severity for IL-6 more than 50 pg/ml on multivariable logistic regression (17). Vultaggio et al. (2020) found that in a cohort of 208 patients, 63 presenting clinical deterioration (defined as oxygen therapy, ICU admission, and death), IL-6 and CRP were predictors of negative outcomes in the first 3 days after hospital admission (18). In another study, maximal IL-6 (>80 pg/mL) and CRP (>97 mg/L) levels before intubation showed the strongest association with the need for mechanical ventilation in a cohort of COVID-19 hospitalized patients (19). These findings along with the results from our study support the use of CRP, a routinely available test, as a reliable predictor of clinical outcome in SARS-CoV-2 positive patients. Whilst, in our cohort, ferritin, D-dimer and LDH were not useful to monitor response to COVID therapies but, as discussed, were a marker of acute disease.

A limitation of the study in predicting severity of COVID-19 was the small sample size available in a low prevalence setting as well as the absence of confounders for most of the control groups (especially NIC). Our unique perspective of comparing IL-6 values among patients with other diseases, in particular those with an associated CS phenotype, provides insight into the underlying pathophysiology of SARS-CoV-2. CS is a somewhat controversial disease state with hyper-cytokinemia including IL-6 as key features (20). In a recent study from the Netherlands, the authors showed that IL-6 values were lower in patients with COVID-19 and acute respiratory distress syndrome (ARDS) when compared to patients with septic shock with ARDS or septic shock without ARDS (21). This was supported by our research, illustrating lower IL-6 values in the SARS-CoV-2 positive patients compared to patients with SAB. The idea that CS is not a prominent feature of severe COVID-19 as previously thought is growing in popularity and is supported by our study. Further examination of novel biomarkers in COVID-19 is required. In the interim, the use of routine tools such as CRP and bedside vital signs may offer the most reliability for clinical prediction.

## Conclusions

• IL-6 levels in COVID-19 are elevated early in disease, although lower compared to other cytokine storm states.
• IL-6 levels follow the response to novel COVID-19 therapies, however do not offer a clinical advantage over C-reactive protein and bedside observations in predicting severe disease.

## Data Availability Statement

The original contributions presented in the study are included in the article**/Supplementary Material**. Further inquiries can be directed to the corresponding author.

## Ethics Statement

The study was approved by the local human research ethics committee (Ref HREC/63201/Austin-20). All patients, their legal representatives or their next of kin provided oral rather than written consent for this study due to COVID-19 restrictions.

## Author Contributions

AC and FJ did the literature review and wrote the manuscript draft. AC and EM were responsible for the laboratory manipulations and data analysis. SV was the statistician responsible for this project. OS, CG, and GD were responsible for patient recruitment, sample follow-up and database entry. NH and JT proposed the study design and manuscript structure. All authors reviewed the current manuscript for important scientific content and made significant contribution to the different sections. All authors contributed to the article and approved the submitted version.

## Funding

This study was supported by unrestricted funding from Austin Health Fundraising. JT was supported by the Austin Medical Research Foundation (AMRF) and by a National Health and Medical Research Council (NHMRC) postgraduate scholarship (GNT 1139902) and Royal Australian College.of Physicians Research Establishment Fellowship.

## Conflict of Interest

The authors declare that the research was conducted in the absence of any commercial or financial relationships that could be construed as a potential conflict of interest.

## Abbreviations

ARDS, acute respiratory distress syndrome; CS, cytokine storm; CRP, C-reactive protein; COVID-19, coronavirus disease 2019; ICU, intensive care unit; IL-6, interleukin-6; NIC, non-infectious condition; SAB, Staphylococcus aureus bacteraemia; SCOVID, suspected COVID-19; SARS-CoV-2, severe acute respiratory syndrome coronavirus 2.
