# Supplementary material for: The Role of Immunological and Clinical Biomarkers to Predict Clinical COVID-19 Severity and Response to Therapy—A Prospective Longitudinal Study
Source: Front Immunol. 2021 Mar 17;12:646095. doi: 10.3389/fimmu.2021.646095 (PMC8009986; doi:10.3389/fimmu.2021.646095)
Supplement: Supplementary file 1 [file Table_1.docx]

The role of immunological and clinical biomarkers to predict clinical COVID-19 severity and response to therapy – A prospective longitudinal study

*Ana Copaescu MD FRCPC^1,2,3^, *Fiona James BBiomedSci^1^, Effie Mouhtouris BSc^1^, Sara Vogrin MBBS MBiostat^4^, Olivia C Smibert MBBS FRACP^5^, Claire L Gordon BMedSci MBBS PhD FRACP^5,6^, George Drewett BMedSci MBBS^5^, **Natasha E Holmes MBBS FRACP PhD^1,5,7^, **Jason A Trubiano BBiomedSci MBBS FRACP PhD^1,5,8, 9^

*Co-first authors

**Co-Senior authors

^1^ Centre for Antibiotic Allergy and Research, Department of Infectious Diseases, Austin Health, Heidelberg, Victoria, Australia

^2^ Clinical Immunology and Allergy, Department of Medicine, McGill University Health Center, Montréal, Canada

^3^ The Research Institute of the McGill University Health Centre, McGill University, Montreal, Canada

^4^ Department of Medicine, St Vincent's Hospital, University of Melbourne, Fitzroy, Victoria, Australia

^5^ Department of Medicine (Austin Health), The University of Melbourne, Heidelberg, Victoria, Australia

^6^ Department of Microbiology and Immunology, The University of Melbourne, Parkville, Victoria, Australia

^7^ Department of Critical Care, Melbourne Medical School, The University of Melbourne, Parkville, Victoria, Australia

^8^ Department of Oncology, Sir Peter MacCallum Cancer Centre, The University of Melbourne, Parkville, Victoria, Australia

^9^ The National Centre for Infections in Cancer, Peter MacCallum Cancer Centre, Parkville, Victoria, Australia

*** Correspondence:**Dr. Ana Copaescu

[ana.copaescu@gmail.com](mailto:ana.copaescu@gmail.com)

**Supplementary Tables**

**Supplementary Table 1** – Longitudinal CRP (mg/L) and IL-6 values (pg/ml) for SARS-CoV-positive patients (n=55). IL-6 serum samples were collected at four timepoints: (1) hospital admission, (2) 24 - 48 hours post-treatment, (3) 7 - 14 days post-treatment, and (4) discharge.

| Patient ID | CRP  Baseline | IL-6  Hospital admission | IL-6  24 - 48 hours post-treatment | IL-6  7 - 14 days post-treatment | IL-6  Discharge |
| --- | --- | --- | --- | --- | --- |
| 1 | 0.9 | 16.4 | n/a | n/a | n/a |
| 2 | 120.0 | 31.6 | n/a | n/a | 7.8 |
| 3 | 94.3 | 30.9 | n/a | n/a | 86.5 |
| 4 | 25.3 | 78.2 | 16.5 | 1.8 | n/a |
| 5 | 12.4 | 21.9 | n/a | n/a | n/a |
| 6 | 237.0 | 85.6 | 72.2 | n/a | n/a |
| 7 | 223.0 | 31.1 | 90.7 | 13.3 | n/a |
| 8 | 59.4 | 37.8 | 165.9 | 27.7 | 9.7 |
| 9 | 22.3 | 77.0 | 17.5 | n/a | n/a |
| 10 | 78.3 | 6.7 | n/a | n/a | 8.9 |
| 11 | 1.6 | 92.5 | n/a | n/a | 114.2 |
| 12 | 1.1 | 28.0 | n/a | n/a | 37.6 |
| 13 | 30.8 | 6.6 | 99.1 | n/a | n/a |
| 14 | 15.0 | 54.8 | n/a | n/a | 26.9 |
| 15 | 144.0 | 741.0 | 1.6 | n/a | n/a |
| 16 | 40.6 | 160.8 | 57.7 | 21.8 | n/a |
| 17 | 135.0 | 16.7 | n/a | 208.2 | n/a |
| 18 | 225.0 | 32.3 | n/a | 4.6 | n/a |
| 19 | 190.0 | 8.0 | -1.4 | n/a | n/a |
| 20 | 8.9 | 87.7 | n/a | n/a | n/a |
| 21 | 112.0 | 207.8 | n/a | 15.3 | n/a |
| 22 | 154.0 | 185.4 | n/a | n/a | 297.1 |
| 23 | 19.4 | 78.2 | n/a | n/a | n/a |
| 24 | n/a | 50.1 | 26.2 | n/a | 20.0 |
| 25 | 201.0 | 161.4 | 5.0 | n/a | 29.3 |
| 26 | 109.0 | 123.2 | 104.4 | n/a | n/a |
| 27 | 106.0 | 6.0 | 2.9 | 206.4 | n/a |
| 28 | 54.0 | 83.2 | n/a | n/a | 136.4 |
| 29 | 49.1 | 0.2 | 3.0 | n/a | -4.3 |
| 30 | 25.0 | 32.5 | n/a | n/a | n/a |
| 31 | 2.9 | 21.4 | 44.2 | 22.5 | 10.0 |
| 32 | 205.0 | 96.8 | n/a | 6.1 | 12.2 |
| 33 | 6.5 | 129.6 | n/a | n/a | n/a |
| 34 | 66.8 | 33.6 | n/a | n/a | 13.1 |
| 35 | 3.2 | 91.4 | n/a | n/a | n/a |
| 36 | n/a | 247.5 | n/a | n/a | 26.7 |
| 37 | 59.2 | 126.4 | n/a | n/a | 103.9 |
| 38 | 43.8 | 196.1 | n/a | n/a | 434.2 |
| 39 | 7.7 | 229.7 | n/a | n/a | n/a |
| 40 | 1.3 | 16.1 | n/a | n/a | n/a |
| 41 | 95.5 | 73.9 | 7.8 | n/a | 9.2 |
| 42 | 18.3 | -0.3 | 2.5 | n/a | n/a |
| 43 | 95.4 | 111.4 | n/a | n/a | n/a |
| 44 | 65.0 | 71.1 | 4.7 | n/a | n/a |
| 45 | 173.0 | 368.6 | n/a | n/a | 2.8 |
| 46 | 125.0 | 231.0 | 22.8 | n/a | 97.2 |
| 47 | 188.0 | 192.4 | n/a | n/a | 59.2 |
| 48 | 6.8 | 49.7 | n/a | n/a | n/a |
| 49 | 50.8 | 50.0 | 35.9 | n/a | 14.5 |
| 50 | 117.0 | 64.1 | 240.7 | 145.3 | n/a |
| 51 | 29.0 | 16.2 | 71.7 | n/a | 31.0 |
| 52 | 142.0 | 108.3 | n/a | n/a | 117.6 |
| 53 | 87.4 | 81.4 | n/a | n/a | 4.7 |
| 54 | 206.0 | 192.8 | 53.4 | n/a | n/a |
| 55 | 248.0 | 56.6 | 0.5 | n/a | 17.4 |

Abbreviations: CRP, C-reactive protein; IL-6, interleukin-6; n/a, non-available data; SARS-CoV-2, severe acute respiratory syndrome coronavirus 2.

**Supplementary Table 2** - IL-6 values (pg/ml) for SARS-CoV-2 positive cohort, age and gender matched with *Staphylococcus aureus* bacteraemia (SAB) and non-infectious condition (NIC) cohorts. SCOVID cohort was recruited concurrently with SARS-CoV-2 cohort but not matched.

| IL-6  SARS-CoV-2 positive | IL-6  SAB | IL-6  NIC | IL-6  SCOVID |
| --- | --- | --- | --- |
| 16.4 | 51.4 | 192.5 | 819.6 |
| 31.6 | 370.4 | 1.1 | 756.7 |
| 30.9 | 173.9 | 42.6 | 40.6 |
| 78.2 | 163.1 | 10.9 | 52.2 |
| 21.9 | 4.5 | 1.7 | 95.5 |
| 85.6 | 19.1 | 68.7 |  |
| 31.1 | 151.8 | 35.8 |  |
| 37.8 | 6.2 | -0.5 |  |
| 77.0 | 255.3 | 26.9 |  |
| 6.7 | 74.6 | -0.9 |  |
| 92.5 | 251.9 | 11.9 |  |
| 28.0 | 3.0 | 14.5 |  |
| 6.6 | 320.8 | 14.4 |  |
| 54.8 | 154.5 | 1.3 |  |
| 741.0 | 90.1 | 37.8 |  |
| 160.8 | 21.8 | 27.2 |  |
| 16.7 | 15.0 | 25.6 |  |
| 32.3 | 96.0 | 7.1 |  |
| 8.0 | 6.0 | 45.5 |  |
| 87.7 | 28.9 | 3.1 |  |
| 207.8 | 313.0 | 7.2 |  |
| 185.4 | 88.7 | 51.7 |  |
| 78.2 | 107.5 | 7.3 |  |
| 50.1 | 197.1 | 122.4 |  |
| 161.4 | 238.7 | 7.9 |  |
| 123.2 | 261.1 | 8.2 |  |
| 6.0 | 60.1 | 39.3 |  |
| 83.2 | 15.2 | 45.6 |  |
| 0.2 | 31.6 | 79.8 |  |
| 32.5 | 13.1 | 23.4 |  |
| 21.4 | 23.9 | 2.4 |  |
| 96.8 | 208.1 | 38.8 |  |
| 129.6 | 89.0 | 2.6 |  |
| 33.6 | 45.7 | 2.1 |  |
| 91.4 | 17.1 | 16.8 |  |
| 247.5 | 111.0 | 10.3 |  |
| 126.4 | 91.4 | 6.4 |  |
| 196.1 | 326.1 | 18.6 |  |
| 229.7 | 12.5 | 23.2 |  |
| 16.1 | 10.4 | 0.0 |  |
| 73.9 | 11.4 | 0.8 |  |
| -0.3 | 218.3 | 8.1 |  |
| 111.4 | 385.2 | 31.7 |  |
| 71.1 | 371.7 | 8.1 |  |
| 368.6 | 1.4 | 23.1 |  |
| 231.0 | 424.8 | 135.6 |  |
| 192.4 | 203.1 | 74.4 |  |
| 49.7 | 236.6 | 12.5 |  |
| 50.0 | 92.8 | 8.1 |  |
| 64.1 | 344.8 | 1.7 |  |
| 16.2 | 457.6 | 12.8 |  |
| 108.3 | 26.9 | 0.6 |  |
| 81.4 | 36.9 | 0.3 |  |
| 192.8 | 246.6 | 3.9 |  |
| 56.6 | 1029.0 | 27.5 |  |

Abbreviations: IL-6, interleukin-6; NIC, non-infectious conditions; SAB, *Staphylococcus aureus* bacteraemia; SARS-CoV-2, severe acute respiratory syndrome coronavirus 2.

**Supplementary Table 3** – Univariable model for prediction of disease severity in SARS-CoV-2 positive patients (n=55)

All variables were measured at baseline. Values reported are estimated odds ratios (95% CI). ORs with a p-value ≤0.5 are in bold and were considered statistically significant.

|  | | Univariable (O_2_ supplement) | | Univariable (ICU admission) | | Univariable (Composite outcome) | |
| --- | --- | --- | --- | --- | --- | --- | --- |
| Variable | N | OR (95% CI) | p - value | OR (95% CI) | p - value | OR (95% CI) | p - value |
| Hemoglobin (g/L) | 55 | 1.01 (0.98, 1.05) | 0.453 | 1.01 (0.97, 1.05) | 0.777 | 1.00 (0.97, 1.04) | 0.907 |
| WCC (x10^9^/L) | 55 | 0.97 (0.88, 1.06) | 0.478 | 1.00 (0.93, 1.07) | 0.905 | 0.98 (0.92, 1.05) | 0.604 |
| Platelet count (x10^9^/L) (increase for 10x x109/L) | 55 | 0.91 (0.83, 1.00) | 0.059 | 1.00 (1.00, 1.01) | 0.412 | 1.00 (0.99, 1.00) | 0.292 |
| Neutrophils (x10^9^/L) | 55 | 1.04 (0.87, 1.25) | 0.673 | 1.14 (0.94, 1.39) | 0.184 | 1.15 (0.94, 1.42) | 0.182 |
| Lymphocytes (x10^9^/L) | 55 | 0.56 (0.18, 1.75) | 0.319 | 0.89 (0.28, 2.84) | 0.849 | 0.42 (0.13, 1.38) | 0.152 |
| Eosinophils (x10^9^/L) (increase for 0.1x x109/L) | 55 | 0.60 (0.15, 2.40) | 0.468 | 0.46 (0.00, 3.9^5) | 0.911 | 0.00 (0.00, 2397.92) | 0.385 |
| Sodium (mmol/L) | 55 | 1.01 (0.91, 1.13) | 0.797 | 1.01 (0.90, 1.14) | 0.822 | 0.98 (0.88, 1.10) | 0.757 |
| Potassium (mmol/L) | 53 | 2.44 (0.66, 9.10) | 0.183 | 1.35 (0.35, 5.17) | 0.663 | 2.50 (0.66, 9.44) | 0.175 |
| Bicarbonate (mmol/L) | 55 | 1.05 (0.96, 1.15) | 0.258 | 0.97 (0.90, 1.04) | 0.433 | 1.03 (0.97, 1.09) | 0.288 |
| Creatinine (μmol/L) | 55 | 1.02 (1.00, 1.05) | 0.038 | 1.01 (0.99, 1.03) | 0.157 | 1.03 (1.00, 1.05) | 0.024 |
| Estimated GFR ♦ | 55 | 0.96 (0.93, 1.00) | 0.034 | 0.98 (0.95, 1.01) | 0.158 | 0.95 (0.92, 0.99) | 0.019 |
| Bilirubin (μmol/L) | 48 | 1.07 (0.95, 1.20) | 0.253 | 1.02 (0.92, 1.14) | 0.674 | 1.03 (0.93, 1.15) | 0.556 |
| ALT (U/L) | 50 | 1.00 (0.98, 1.02) | 0.804 | 1.00 (0.98, 1.02) | 0.98 | 1.00 (0.98, 1.01) | 0.664 |
| AST (U/L) | 13 | 1.00 (0.98, 1.02) | 0.733 | 1.01 (0.99, 1.03) | 0.543 | 1.00 (0.98, 1.02) | 0.733 |
| GGT (U/L) | 55 | 1.01 (1.00, 1.02) | 0.158 | 1.01 (1.00, 1.02) | 0.138 | 1.01 (1.00, 1.02) | 0.175 |
| ALP (U/L) | 55 | 0.99 (0.97, 1.01) | 0.182 | 0.99 (0.97, 1.01) | 0.372 | 0.99 (0.97, 1.01) | 0.198 |
| Albumin (g/L) | 53 | 0.89 (0.79, 1.00) | 0.046 | 0.84 (0.74, 0.96) | 0.011 | 0.81 (0.70, 0.93) | 0.004 |
| CRP (mg/L) (increase for 10 mg/L) | 53 | 1.13 (1.04, 1.23) | 0.006 | 1.01 (1.00, 1.02) | 0.014 | 1.01 (1.00, 1.02) | 0.003 |
| Ferritin (μg/L) (increase for 100 (μg/L) | 50 | 1.11 (1.00, 1.24) | 0.05 | 1.00 (1.00, 1.00) | 0.011 | 1.00 (1.00, 1.00) | 0.06 |
| LDH (units/L) (increase for 100 units/L) | 33 | 1.59 (0.93, 2.69) | 0.087 | 1.01 (1.00, 1.02) | 0.017 | 1.00 (1.00, 1.01) | 0.122 |
| D-dimer (ng/mL) (increase for 100 ng/mL) | 53 | 1.01 (0.98, 1.03) | 0.555 | 1.00 (1.00, 1.00) | 0.304 | 1.00 (1.00, 1.00) | 0.672 |
| IL-6 levels (pg/ml) | 55 | 0.99 (0.95, 1.04) | 0.829 | 0.99 (0.98, 1.00) | 0.097 | 1.00 (1.00, 1.00) | 0.996 |

Abbreviations: ALP, alkaline phosphatase; ALT, alanine aminotransferase; AST, aspartate transaminase; CRP, C-Reactive protein; GFR, glomerular filtration rate; GGT, gamma-glutamyl transferase; IL-6, Interleukin-6 ; LDH, lactate dehydrogenase; SARS-CoV-2, severe acute respiratory syndrome coronavirus 2; WCC, white cell count.

Ψ Patients that required supplemental O_2_ continuously for more than 24 hours during their admission.

♦ Estimated GFR was calculated using Chronic Kidney Disease Epidemiology Collaboration (CKD-EPI), units: ml/min/1.73.

**Supplementary Table 4 –** Univariable model for prediction of each outcome: requirement for supplemental oxygen, ICU admission and a composite of both outcomes in SARS-CoV-2 positive patients (n=55). Values reported are estimated odds ratios (95% CI). Those with a p-value <0.1 are in bold and were included in the multivariable model.

|  | Supplemental O_2_ | | ICU Admission | | Composite | |
| --- | --- | --- | --- | --- | --- | --- |
| Variable | OR (95% CI) | p-value | OR (95% CI) | p-value | OR (95% CI) | p-value |
| Age (years) | 1.05 (1.01, 1.09) | 0.009 | 1.02 (0.99, 1.06) | 0.159 | 1.05 (1.01, 1.09) | 0.006 |
| Sex (male vs female) | 2.43 (0.80, 7.33) | 0.115 | 1.16 (0.36, 3.68) | 0.806 | 2.31 (0.77, 6.88) | 0.133 |
| Charlson comorbidity index (age adjusted) | 1.49 (0.99, 2.24) | 0.054 | 1.03 (0.69, 1.54) | 0.881 | 1.55 (1.03, 2.35) | 0.038 |
| Hypertension or cardiac disease | 4.93 (1.54, 15.79) | 0.007 | 3.10 (0.95, 10.11) | 0.061 | 5.33 (1.63, 17.44) | 0.006 |
| Chronic respiratory disease | 3.66 (0.96, 13.86) | 0.056 | 2.42 (0.67, 8.77) | 0.18 | 3.00 (0.80, 11.31) | 0.105 |
| Diabetes | 3.33 (0.96, 11.63) | 0.059 | 1.76 (0.51, 6.10) | 0.374 | 4.12 (1.12, 15.25) | 0.034 |
| Immunosuppression | 1.91 (0.29, 12.44) | 0.499 | 3.86 (0.58, 25.60) | 0.162 | 1.62 (0.25, 10.58) | 0.611 |
| ACEI/ARB use | 2.84 (0.63, 12.80) | 0.174 | 1.14 (0.25, 5.23) | 0.863 | 2.38 (0.53, 10.70) | 0.258 |
| Fever | 1.00 (0.27, 3.77) | 1 | 0.73 (0.18, 2.94) | 0.662 | 0.76 (0.20, 2.87) | 0.686 |
| Malaise/myalgia | 0.85 (0.29, 2.49) | 0.765 | 0.73 (0.23, 2.32) | 0.599 | 0.60 (0.20, 1.76) | 0.351 |
| Diarrhoea | 0.91 (0.28, 2.93) | 0.871 | 1.02 (0.29, 3.59) | 0.972 | 1.05 (0.33, 3.37) | 0.931 |
| Shortness of breath | 3.67 (0.88, 15.25) | 0.074 | 3.06 (0.60, 15.65) | 0.18 | 2.72 (0.72, 10.25) | 0.138 |
| Cough, coryza or sore throat | 0.54 (0.17, 1.75) | 0.306 | 1.50 (0.40, 5.58) | 0.545 | 0.67 (0.21, 2.15) | 0.497 |
| Other symptoms | 0.29 (0.05, 1.52) | 0.143 | n/a | n/a | 0.24 (0.04, 1.28) | 0.095 |
| COVID-MATCH65 Scoring Assessment Φ | 1.49 (1.04, 2.13) | 0.029 | 1.19 (0.85, 1.67) | 0.313 | 1.44 (1.02, 2.05) | 0.04 |
| Temperature (tympanic) | 2.03 (1.17, 3.50) | 0.011 | 1.38 (0.82, 2.31) | 0.222 | 1.64 (0.99, 2.71) | 0.054 |
| Respiratory rate | 1.18 (1.06, 1.31) | 0.002 | 1.17 (1.06, 1.30) | 0.002 | 1.22 (1.08, 1.37) | 0.002 |
| SpO_2_ | 0.62 (0.47, 0.82) | 0.001 | 0.81 (0.69, 0.96) | 0.017 | 0.67 (0.52, 0.86) | 0.001 |
| Pulse rate | 1.03 (0.99, 1.07) | 0.112 | 1.04 (1.00, 1.09) | 0.047 | 1.03 (0.99, 1.07) | 0.139 |
| Systolic Blood Pressure | 0.99 (0.97, 1.02) | 0.462 | 0.98 (0.95, 1.01) | 0.2 | 0.98 (0.96, 1.01) | 0.228 |
| Diastolic Blood Pressure | 0.97 (0.93, 1.01) | 0.192 | 0.97 (0.93, 1.02) | 0.251 | 0.97 (0.92, 1.01) | 0.131 |

Abbreviations: ACEI, angiotensin-converting-enzyme inhibitors; ARB, angiotensin II receptor blockers; SARS-CoV-2, severe acute respiratory syndrome coronavirus 2; SpO2, oxygen saturation; n/a, not applicable

Φ COVID-MATCH65 Score is an internally derived clinical decision rule that has a high sensitivity (92.6%) and NPV (99.5%) for SARS-CoV-2 and could be used to aid COVID-19 risk assessment and resource allocation for SARS-CoV-2 diagnostics. The resulting score ranges from 1 to 6.5 points with scores ≤ 1 representing low risk of a positive test (16).
